# Simvastatin treatment promotes proliferation of human dental pulp stem cells via modulating PI3K/AKT/miR‐9/KLF5 signalling pathway

**DOI:** 10.1111/jcmm.16973

**Published:** 2021-11-19

**Authors:** Jing‐hui Wang, Dang‐en He

**Affiliations:** ^1^ General Department Qingdao Stomatological Hospital Qingdao China; ^2^ Stomatology Department Yangling Demonstration Zone Hospital Yangling China

**Keywords:** AKT, KLF5, miR‐9, PI3K, proliferation, pulp stem cells, simvastatin

## Abstract

Simvastatin serves as an effective therapeutic potential in the treatment of dental disease via alternating proliferation of dental pulp stem cells. First, western‐blot and real‐time quantitative PCR were used to detect the effect of simvastatin or LY294002 on the expression levels of AKT, miR‐9 and KLF5, or determine the effect of miR‐9. Simvastatin, KLF5 and AKT significantly enhanced the proliferation of pulp stem cells, whilst this effect induced by simvastatin was suppressed by LY294002, AKT siRNA, KLF5 siRNA and miR‐9, and simvastatin dose‐dependently upregulated the expression of PI3K. Furthermore, simvastatin upregulated PI3K and p‐AKT expression in a concentration‐dependent manner. LY294002 abrogated the upregulation of p‐AKT expression levels induced by simvastatin, and LY294002 induced the miR‐9 expression and simvastatin dose‐dependently inhibited the expression of miR‐9, by contrast, LY294002 reduced the KLF5 expression and simvastatin dose‐dependently promoted the expression of KLF5. And using computational analysis, KLF5 was found to be a candidate target gene of miR‐9, and which was further verified using luciferase assay. Finally, the level of KLF5 in cells was much lower following the transfection with miR‐9 and KLF5 siRNA, and the level of AKT mRNA in cells was significantly inhibited after transfection with AKT siRNA than control. These findings suggested simvastatin could promote the proliferation of pulp stem cells, possibly by suppressing the expression of miR‐9 via activating the PI3K/AKT signalling pathway, and the downregulation of miR‐9 upregulated the expression of its target gene, KLF5, which is directly responsible for the enhanced proliferation of pulp stem cells.

## 1 INTRODUCTION

As the only vascularized tissue in the tooth, the dental pulp is embedded in a structure of dentin rich in minerals. Dental infections or pulp trauma that is often characterized as pulpitis can impair the overall health of the tooth.^1^ Responding to certain damages to the pulp, reparative dentin occurs.^2^ The definition of a correlation between intrinsic repair and defect size of dental pulp exposure is of critical significance to the regeneration of dental pulp.^3^ There is a subpopulation of cells with the phenotypic features of stem cells in human dental pulp, as revealed by their expression of multiple mesenchymal stem cell surface markers, multilineage differentiation, strong self‐renewal and proliferative potentials.^4^ Dental pulp cells (DPCs) can undergo differentiation into odontoblast‐like cells and form mineral structures like dentine in vitro and in vivo.^5^ They are promising sources of cellular therapy for endodontic regeneration. Okamoto et al described that DPSCs treated by Simvastatin (SIM) revealed enhanced mineralized tissue formation and promoted odontogenic differentiation.^6^ Moreover, Karanxha et al and Min et al.^7^, ^8^ indicated that SIM enhances odontogenesis in human dental pulp cells. It has been predicted that SIM could enhance mineralization and DPSC differentiation and hence may have promising application in the regeneration of pulp.^9^ A range of studies conducted in animal models has shown that DPSC transplantation in the root canal results in the regeneration of pulp.^10^

As zinc finger‐containing transcription factors, Krüppel‐like factors (KLFs) are associated with several cellular processes such as apoptosis, cell cycle, proliferation and differentiation.^11^ A recent study has shown that KLF5 was necessary for the transformation and survival of intestinal epithelial cells.^12^ They also revealed an elevation in apoptotic bodies and decreased cell proliferation upon KLF5 deletion from the intestinal stem cells dependent on nuclear localization of β‐catenin.^12^ They found that KLF5 was a modulator of neoplastic and homeostatic proliferation in intestinal epithelial cells.^12^ It has been shown that KLF5 may be associated with mineralization of both dentine and enamel matrices and involved in the early stages of tooth development.^13^ Providing these earlier results, it was postulated that KLF5 may involve in the differentiation of odontoblast during the formation of reparative dentine. Moreover, KLF5 can enhance the activity of b‐catenin, which has been suggested to promote the differentiation of odontoblast of DPCs.^14^, ^15^

As small non‐coding RNAs of 19–25 nucleotides in length, microRNAs (miRNAs) are identified to control a range of protein‐coding genes either in animals or plants. The first miRNA is known as lin‐4 that regulated timing of development in Caenorhabditis elegans was found by two individual groups in 1993.^16^ Consequently, a number of miRNAs have been involved in various cellular processes such as metabolism, stress response, stem cell renewal, embryonic development, cell proliferation, differentiation and apoptosis.^17^

KLF5 has been shown to regulate the proliferation of pulp stem cells, and simvastatin was shown to activate the PI3K and AKT signalling pathway and down‐regulate the expression of miR‐9.^9^, ^18^, ^19^ Moreover, as a therapeutic method for various diseases, the homing of bone marrow‐derived mesenchymal stem cells (BMSCs) to injury sites could be promoted by simvastatin via the modulation of the PI3K/AKT/miR‐9 pathway. Moreover, KLF5 was also found to be a potential target of miR‐9 in the online miRNA database. In this study, we aimed to study the effect of Simvastatin on the control of dental pulp cells regeneration as well as the underlying molecular mechanisms which included miR‐9 and KLF5.

## 2 MATERIALS AND METHODS

### 2.1 Human pulp stem cells isolation and culture

Pulp stem cells were isolated from the resected tissue samples from recruited patients as described previously.^9^ DMEM (Dulbecco's modified Eagle's medium) including 10% FBS (fetal bovine serum), 100 μg/ml streptomycin and 100 U/ml penicillin G was used to maintain pulp stem cells. We flushed and then cultured dental marrow, and PBS (phosphate‐buffered saline) was used to wash the sample to remove the haematopoietic cells and changed the medium at 48 h. Cells from passages 3 to 5 were used in the present experiments. Ethical approval was obtained from the Ethical committee of Yangling Demonstration Zone Hospital and signed written informed consent was obtained before the initiation of this study.

### 2.2 RNA isolation and real‐time PCR

TRIzol reagent (Invitrogen) was used to extract the total RNA from the tissue samples and pulp cells according to the manufacturer's recommendation. BioPhotometer plus from Eppendorf AG (Hamburger, Germany) was used to check the RNA quantity and quality based on the description by the supplier. ReverTra Ace qPCR RT Kit (TOYOBO) was used to synthesize the cDNA from RNA extracted to detect the expression of miR‐9 and PI3K, AKT and KLF5 mRNA. M‐MLV reverse transcriptase (Invitrogen) was used to reverse transcribe RNA extracted based on the manufacturer's recommendation. ABI 7500 real‐time PCR system (AppliedBiosystems) and real‐time PCR Master mix kit (TOYOBO) were used to quantify the expression of miR‐9 (Forward: 5′‐ ATCCAGTGCGTGTCGTG −3′; Reverse: 5′‐ TGCTTCTTTGGTTATCTAGC −3′) and PI3K (Forward: 5′‐ CGAGAGTGTCGTCACAGTGTC −3′; Reverse: 5′‐ TGTTCGCTTCCACAAACACAG −3′), AKT (Forward: 5′‐ CCTCCACGACATCGCACTG −3′; Reverse: 5′‐ TCACAAAGAGCCCTCCATTATCA −3′) and KLF5 (Forward: 5′‐ CCTGGTCCAGACAAGATGTGA −3′; Reverse: 5′‐ GAACTGGTCTACGACTGAGGC −3′) mRNA, and snRNA U6 (Forward: 5′‐ CTCGCTTCGGCAGCACA −3′; Reverse: 5′‐ AACGCTTCACGAATTTGCGT −3′) and GAPDH (Forward: 5′‐AGCCTCAAGATCATCAGCAATG −3′; Reverse, 5′‐TGTGGTCATGAGTCCTTCCACG‐3′) were served as the internal control. The PCR reaction was performed at 95℃ at 70 s for initial denaturation, subsequently 48 cycles of 95℃ for 15 s, 60℃ for 30 s, 72℃ for 70 s. The 2^−ΔΔCT^ method was used to detect the relative expression of miR‐9 and PI3K, AKT and KLF5 mRNA normalized to the expression of snRNA U6. All tests were performed thrice.

### 2.3 Cell transfection

When the cells reached 60%–70% confluence, lipofectamine 2000 (Invitrogen) was used to transfect the cells with miR‐9 mimics, or the siRNA against KLF5 and negative control (Gene Pharma) in accordance with the manufacturer's instruction. Three independent experiments were carried out.

### 2.4 Cell proliferation assay

Cell Counting Kit‐8 (Dojindo Laboratories, Kumamoto, Japan) was used to examine the proliferation ability of pulp cells transfected with miR‐9 mimics or inhibitors in accordance with the manufacturer's instruction. In brief, 10^3^/well were cultured in a 48‐well plate for 0, 24, 48, 72, and 96 h, and then added 10‐μl CCK‐8 solution reagent into each well. And a microplate reader was used to calculate the proliferation based on the absorbance value at 450 nm. Each experiment was run thrice.

### 2.5 Luciferase assay

The chromosome segment including wild‐type 3′UTR of KLF5 was amplified by PCR, and the PCR products were inserted into pMIRGLO‐REPORT luciferase vector (Ambion), and mutations were introduced into the miR‐9 binding site which was named pMIRGLO‐ KLF5‐3’UTR MT and pMIRGLO‐KLF5‐3′UTR‐WT, respectively. The Lipofectamine 2000 (Invitrogen, Life Technologies) was used to transfect the pulp cells with Renilla constructs, pMIRGLO‐ KLF5‐3’UTR MT, pMIRGLO‐ KLF5‐3′UTR‐WT with or without miR‐9 mimics following the supplier's instruction. The Dual‐Luciferase Assay system (Promega) was used to detect the luciferase activity 48 h post‐transfection based on the manufacturer's introductions. Renilla constructs were used as internal controls and normalized the luciferase activity. Each test was performed thrice.

### 2.6 Western blot analysis

RIPA lysis buffer (Sigma‐Aldrich) including 1% sodium deoxycholate, 1% Triton X‐100, 0.1% SDS, 50 mM Tris (pH 7.4), 5 mM EDTA (pH 8.0), 2 mM NaF and phenylmethylsulfonylfluoride protease inhibitor, was used to isolate protein from pulp cells and tissue samples following the manufacturer's guideline. Twelve per cent SDS‐polyacrylamide gels were used to isolate the protein (30 μg) extracted, and then transferred to 0.22‐μm nitrocellulose filter membranes (Millipore). The non‐fat dry milk (Merck) was used to block the membranes for 60 min, the primary antibody against PI3K(1:1,000; Santa Cruz Biotechnology), p‐AKT (1:1,000; Santa Cruz Biotechnology) and KLF5 (1:2,000; Santa Cruz Biotechnology) and anti‐β‐actin at a dilution of 1:10000 (Santa Cruz Biotechnology) were used to hybridize the membranes at 4℃ for 12 h. Horseradish peroxidase‐linked anti‐rabbit secondary antibodies (1:15000, Santa Cruz Biotechnology) were used to detect the primary antibodies at room temperature for 60 min. The enhanced chemiluminescent substrate (Pierce) was used to detect the levels of phosphor‐proteins and proteins. Each experiment was performed thrice.

### 2.7 Statistical analysis

SPSS software (version 10.0) was used to perform the statistical analyses. All data were shown as the means ± SEM (standard error means). The student's *t*‐test was used to detect the statistical difference, and the Bonferroni test was used to analyse the SEM of data by use of variance (ANOVA). It was considered significant when the *p* value was < 0.05 in all of the significance analyses.

## 3 RESULTS

### 3.1 Simvastatin promoted the proliferation of pulp stem cells

**FIGURE 1:**
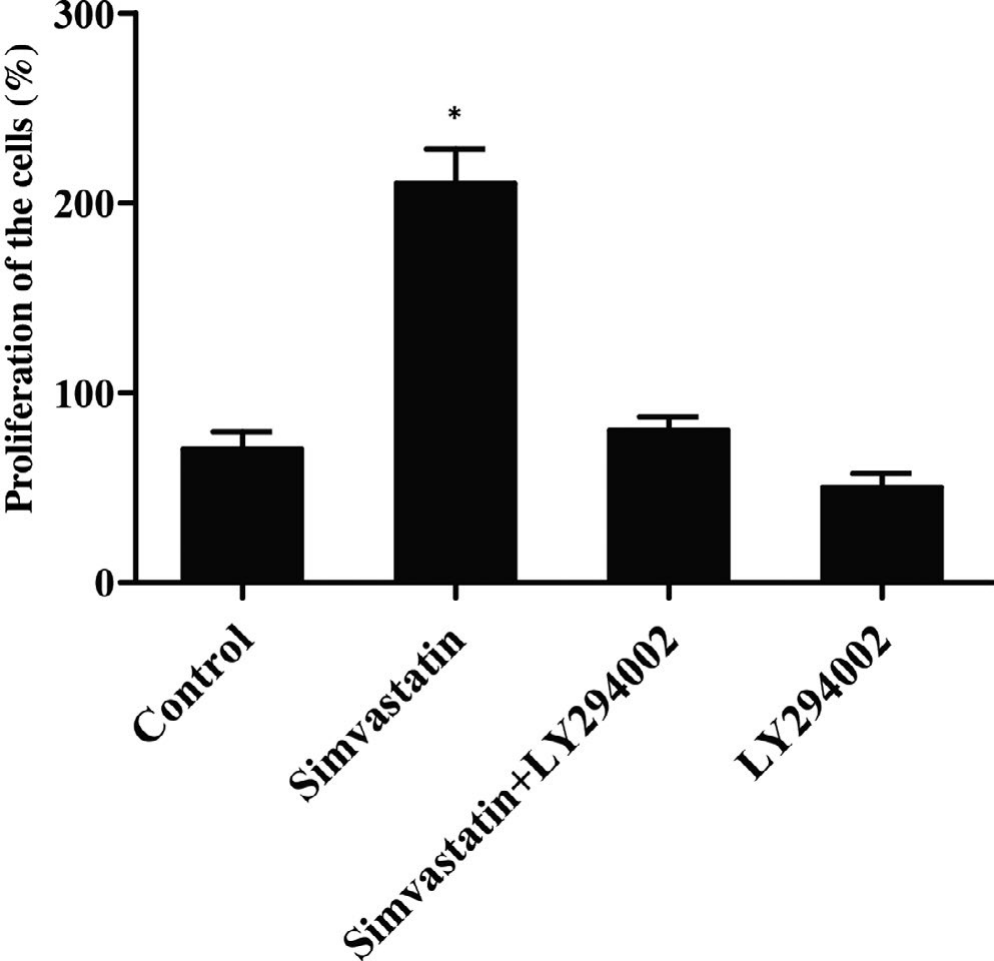
Simvastatin enhanced the proliferation of pulp stem cells (Asterisk *p* value < 0.05 vs. control group, untreated cells were referred to a control group)

In order to examine the effect of simvastatin on the survival of pulp stem cells, CCK‐8 assay was investigated, as shown in Figure 1, the cells treated with 1‐um simvastatin for 48 h substantially enhanced proliferation than control. On the contrary, cells treated with LY294002, an inhibitor of PIK3 substantially inhibited proliferation compared with control; furthermore, the cells exposed to simvastatin and LY294002 showed no obvious difference in proliferation with control, suggesting that simvastatin significantly enhanced proliferation of pulp stem cells, but this effect was completely abolished by LY294002.

### 3.2 Simvastatin effect on expression levels of PI3K, AKT and p‐AKT (Ser473)

**FIGURE 2:**
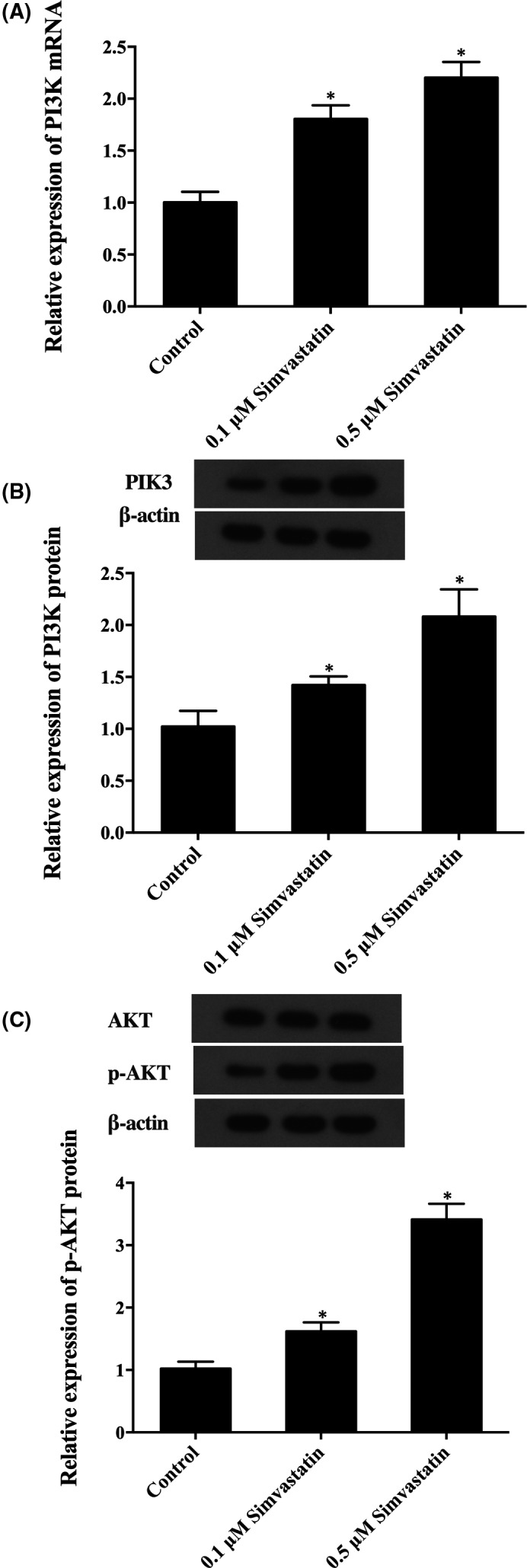
The effect of simvastatin on expression levels of PI3K, AKT and p‐AKT (Asterisk *p* value < 0.05 vs. control group, untreated cells were referred to a control group). (A) PI3K mRNA level of cells treated with different doses of simvastatin. (B) PI3K protein level of cells treated with different doses of simvastatin. (C) p‐AKT and AKT protein level of cells treated with different doses of simvastatin

To confirm, the above proliferation increase was regulated by PI3K, an important protein factor in PIK3/AKT signalling pathway. Western‐blot analysis and real‐time quantitative PCR were used to measure the level of PI3K in cells after treatment with different concentrations of simvastatin, as shown in Figure 2A,B, simvastatin dose‐dependently up‐regulated the expression of PI3K mRNA (Figure 2A) and protein (Figure 2B) compared with those in the control group. Moreover, to further confirm whether simvastatin induced the expression of AKT or enhanced AKT phosphorylation at Ser473, western‐blot was performed to detect the levels of AKT and p‐AKT after treating with different concentrations of simvastatin (0.1 μM and 0.5μM). As shown in Figure 2C, the p‐AKT expression level of cells treated with 0.1 μM simvastatin was much higher than control, whilst the p‐AKT level of cells treated with 0.5 μM simvastatin was even higher compared to that in 0.1 μM simvastatin treatment groups. Meanwhile, the levels of AKT of cells treated with different concentrations of simvastatin (0.1 μM and 0.5 μM) were comparable with control, indicating that simvastatin upregulated PI3K and p‐AKT expression under a concentration‐dependent manner, whilst simvastatin had no effect on the level of AKT, leading to the hypothesis that expression of PI3K might be affected by p‐AKT expression.

### 3.3 Simvastatin up‐regulated the expression of PI3K and p‐AKT

**FIGURE 3:**
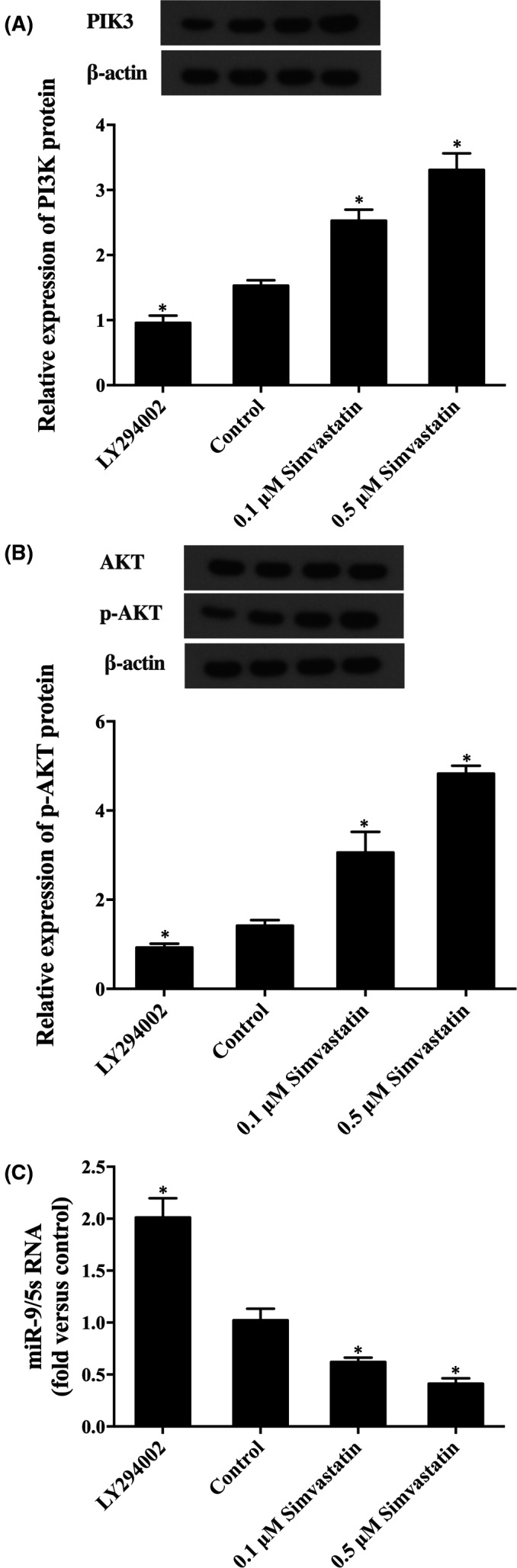
The effect of LY294002 on the simvastatin‐regulated expressions of PI3K, p‐AKT, AKT and miR‐9 (Asterisk *p* value < 0.05 vs. control group, untreated cells were referred to a control group). (A) PI3K protein expression levels in cells exposed to simvastatin and/or LY294002. (B) p‐AKT and AKT protein expression levels in cells exposed to simvastatin and/or LY294002. (C) MiR‐9 expression levels in cells exposed to simvastatin and/or LY294002

To verify the above hypothesis, LY294002 and simvastatin were used to treat the cells, and the levels of PI3K, p‐AKT and AKT were detected using western‐blot. As shown in Figure 3, simvastatin dose‐dependently upregulated the expression of PI3K protein (Figure 3A) and p‐AKT (Figure 3B), and LY294002 completely abrogated the simvastatin‐induced up‐regulation of PI3K and p‐AKT. Meanwhile, LY294002 and simvastatin had no effect on the expression of AKT protein (Figure 3B), which validated that simvastatin caused the over‐expression of PI3K through the PI3K/AKT pathway. To further confirm whether the expression of miR‐9 is caused by PI3K/AKT pathway, the level of miR‐9 in cells treated with LY294002 and different concentrations of simvastatin was detected by real‐time PCR, as shown in Figure 3C, the miR‐9 expression level in cells treated with 0.1 μM simvastatin was evidently lower than the untreated cells, and the miR‐9 level in cells treated with 0.5 μM simvastatin was even lower in comparison with that in 0.1 μM simvastatin treatment groups. Meanwhile, LY294002 evidently increased the level of miR‐9.

### 3.4 KLF5 was the direct target of miR‐9

**FIGURE 4:**
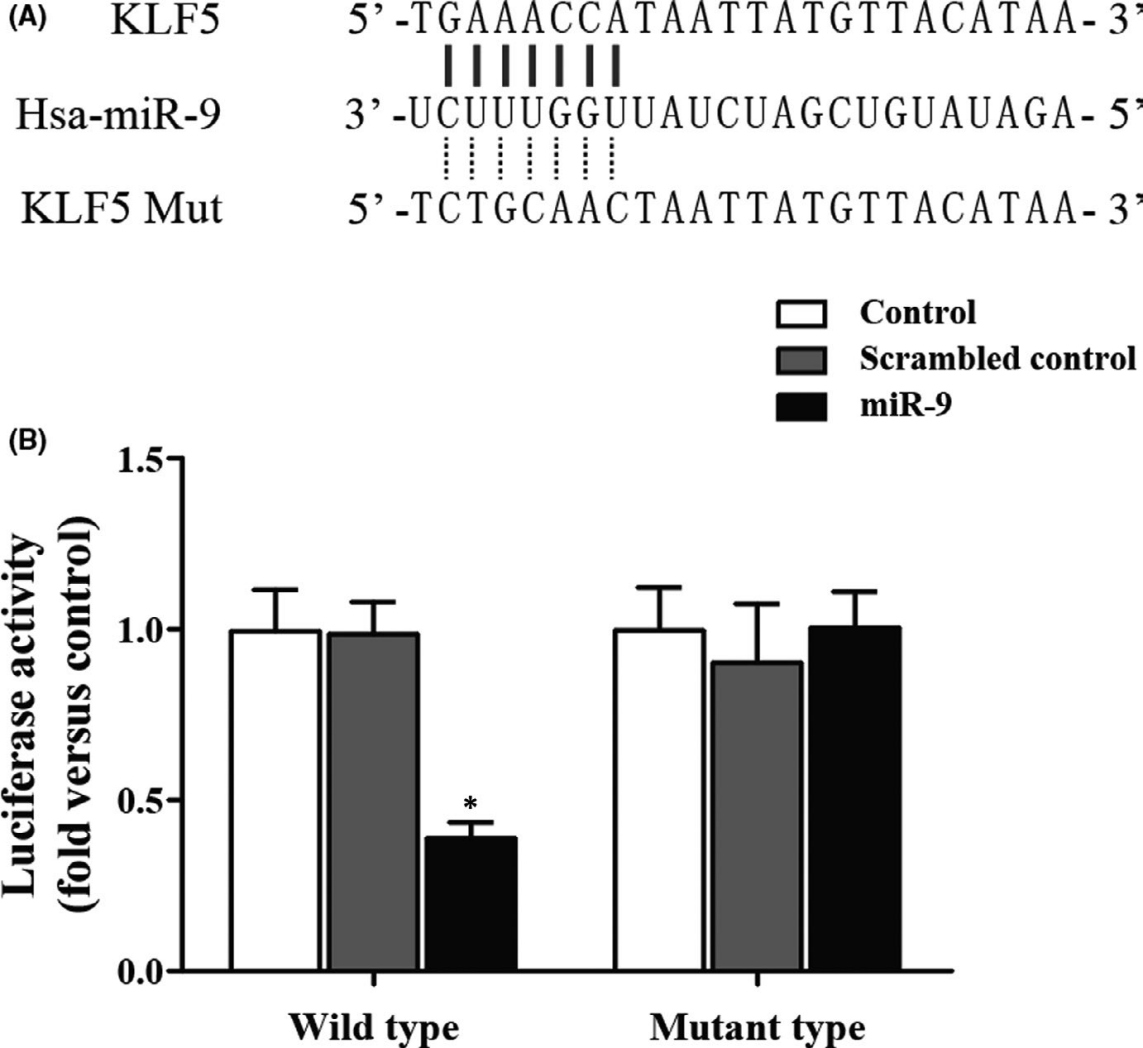
The correlations between KLF5 and miR‐9 (mut: mutant). (A) Schematic comparison between miR‐9 and the “seed sequence” in the 3’UTR of KLF5. (B) Luciferase activities in the miR‐9 overexpressing cells transfected with wild‐type or mutant 3’UTR of KLF5 (Asterisk *p* value < 0.05 vs. control group, cells treated with control miRNAs were referred as the control group)

Based on the result of TargetScan 5.2, an online miRNA prediction tool miRanda, KLF5 was predicted to be the direct target of miR‐9 with the binding site located on 3’UTR (Figure 4A). To further confirm whether KLF5 was a direct downstream target of miR‐9, we performed a luciferase reporter assay. As shown in Figure 4B, the miR‐9 mimic obviously inhibited the wild‐type KLF5 3′UTR luciferase activity. On the contrary, miR‐9 mimic failed to affect the luciferase activity of mutant KLF5 3′UTR compared to that in scramble control, which suggested that miR‐9 negatively mediated the expression of KLF5 via binding to the sequence located on the 3′‐ UTR of KLF5.

### 3.5 Simvastatin‐enhanced expression of KLF5

**FIGURE 5:**
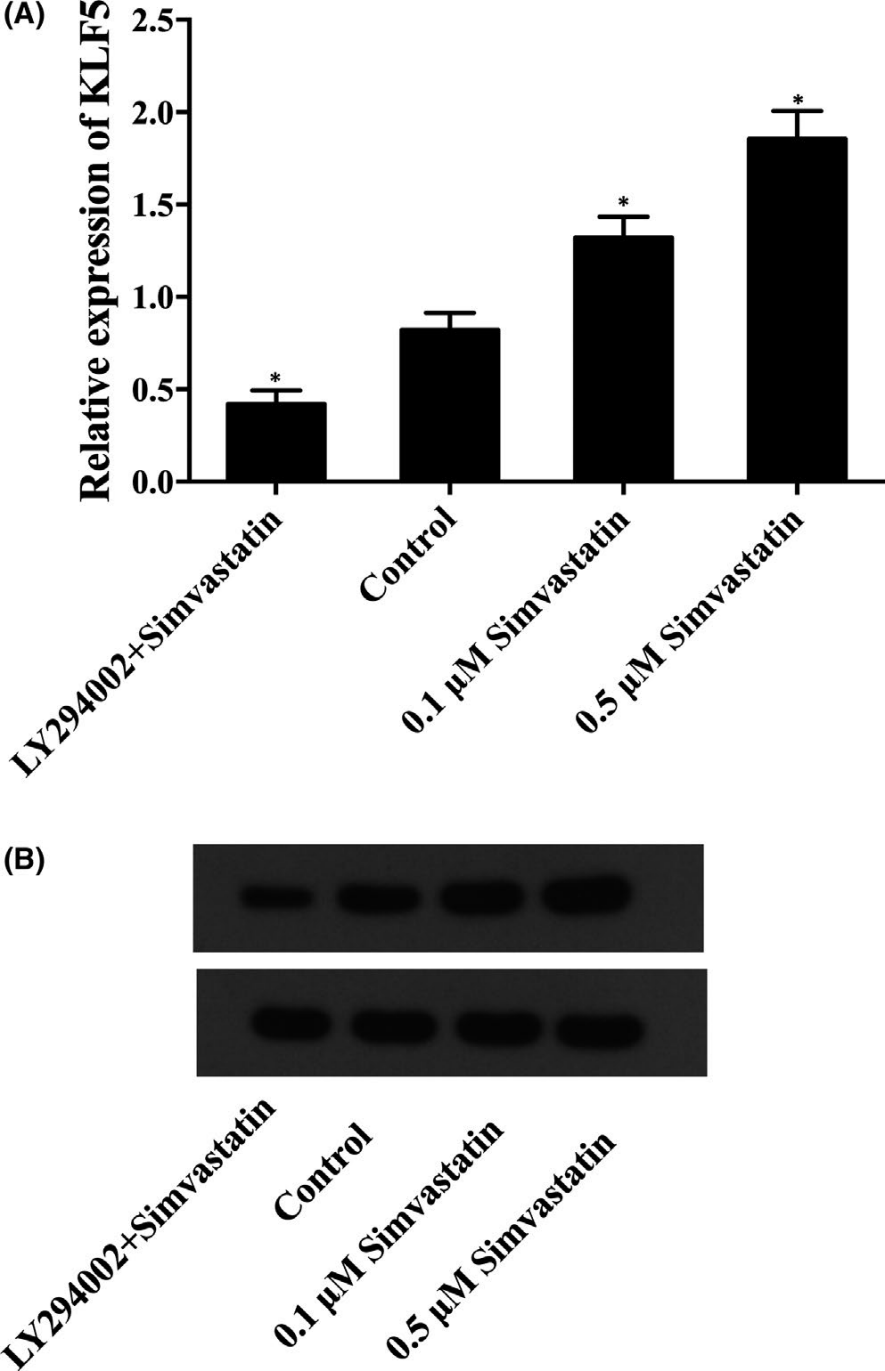
The effect of simvastatin on the expression levels of KLF5 (asterisk *p* value < 0.05 vs. control group, untreated cells were referred as the control group). (A) KLF5 mRNA level of cells treated with different doses of simvastatin or LY294002. (B) KLF5 protein level of cells treated with different doses of simvastatin or LY294002

Because miR‐9 negatively mediated the expression of KLF5, the effect of simvastatin on the KLF5 level exhibited an opposite trend compared with the effect of simvastatin on the miR‐9 level. Western‐blot analysis and real‐time quantitative PCR were performed to prove this hypothesis, and the levels of KLF5 in cells following treatment with LY294002 and different concentrations of simvastatin were examined. As shown in Figure 5, the KLF5 expression level of cells treated with 0.1 μM simvastatin was evidently higher than the control, and the KLF5 level of cells treated with 0.5 μM simvastatin was even higher in comparison with that in 0.1 μM simvastatin treatment groups. Meanwhile, LY294002 evidently decreased the level of KLF5. These data indicated that simvastatin dose‐dependently upregulated KLF5.

### 3.6 Exogenous expression of miR‐9 suppressed the expression of KLF5

**FIGURE 6:**
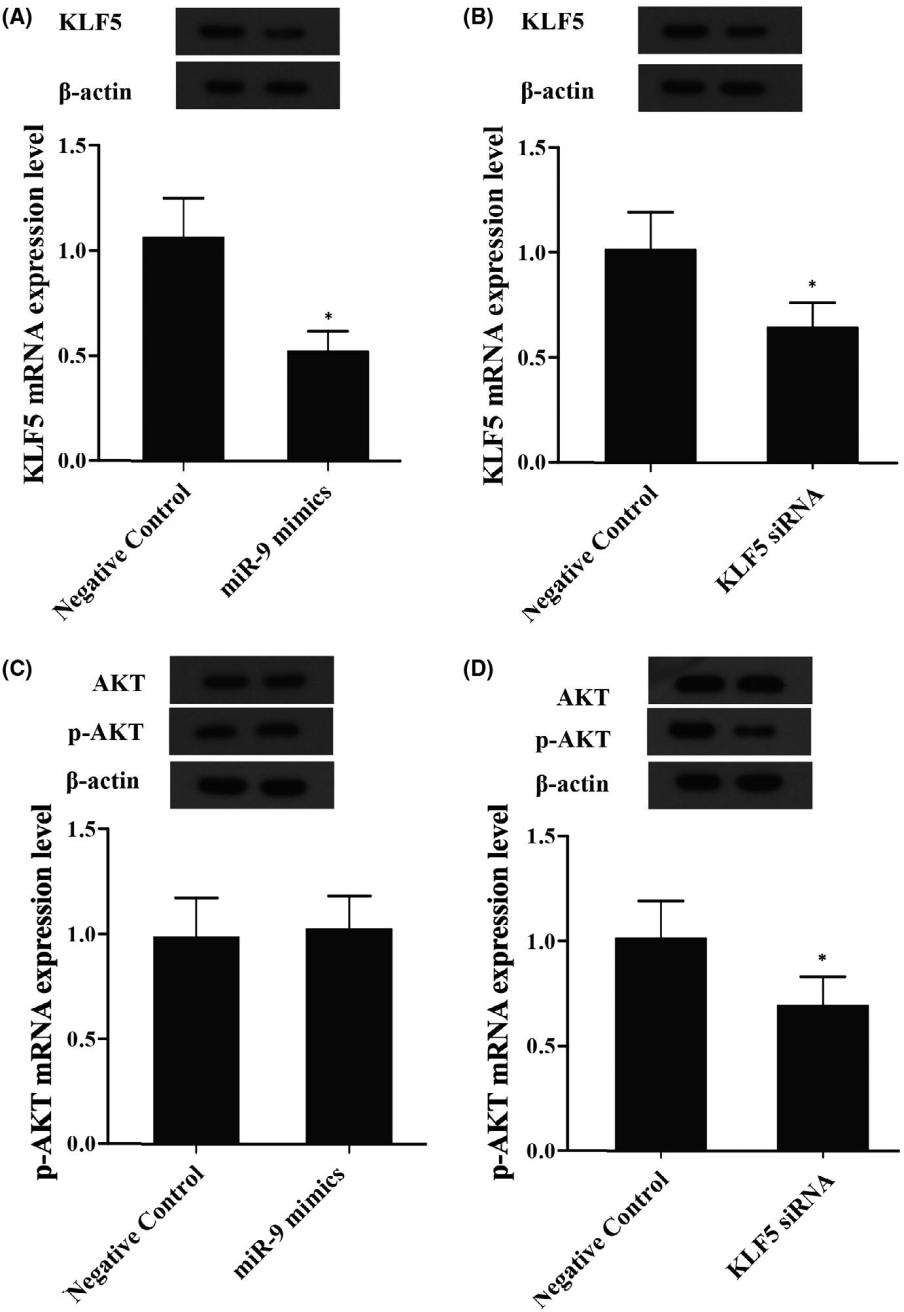
The effect of miR‐9 on levels of KLF5 and AKT (Asterisk *p* value < 0.05 vs. negative control group, untreated cells were referred as the control group). (A) The levels of KLF5 mRNA and protein in cells transfected with miR‐9 mimic. (B) The levels of KLF5 mRNA and protein in cells transfected with KLF5 siRNA. (C) The levels of AKT mRNA and protein in cells transfected with miR‐9 mimic. (D) The levels of AKT mRNA and protein in cells transfected with AKT siRNA

MiR‐9 mimic, KLF5 siRNA and AKT siRNA together with scrambled control were transfected into the pulp stem cells, and we examined the mRNA and protein expression levels of KLF5 and AKT using western‐blot and real‐time PCR and found that obvious decrease in mRNA and protein (Figure 6A,B) levels of KLF5 in cells following the introduction of miR‐9 mimic (Figure 6A) and KLF5 siRNA (Figure 6B) compared with control, and the level of AKT of cells transfected with AKT siRNA was apparently downregulated than control (Figure 6D), and level of AKT of cells transfected with miR‐9 mimic showed no significant difference with control (Figure 6C), indicating that KLF5 was the target gene of miR‐9, which suppressed the expression of KLF5, and miR‐9 had no effect on the expression of AKT.

### 3.7 Simvastatin promoted the proliferation of pulp stem cells

**FIGURE 7:**
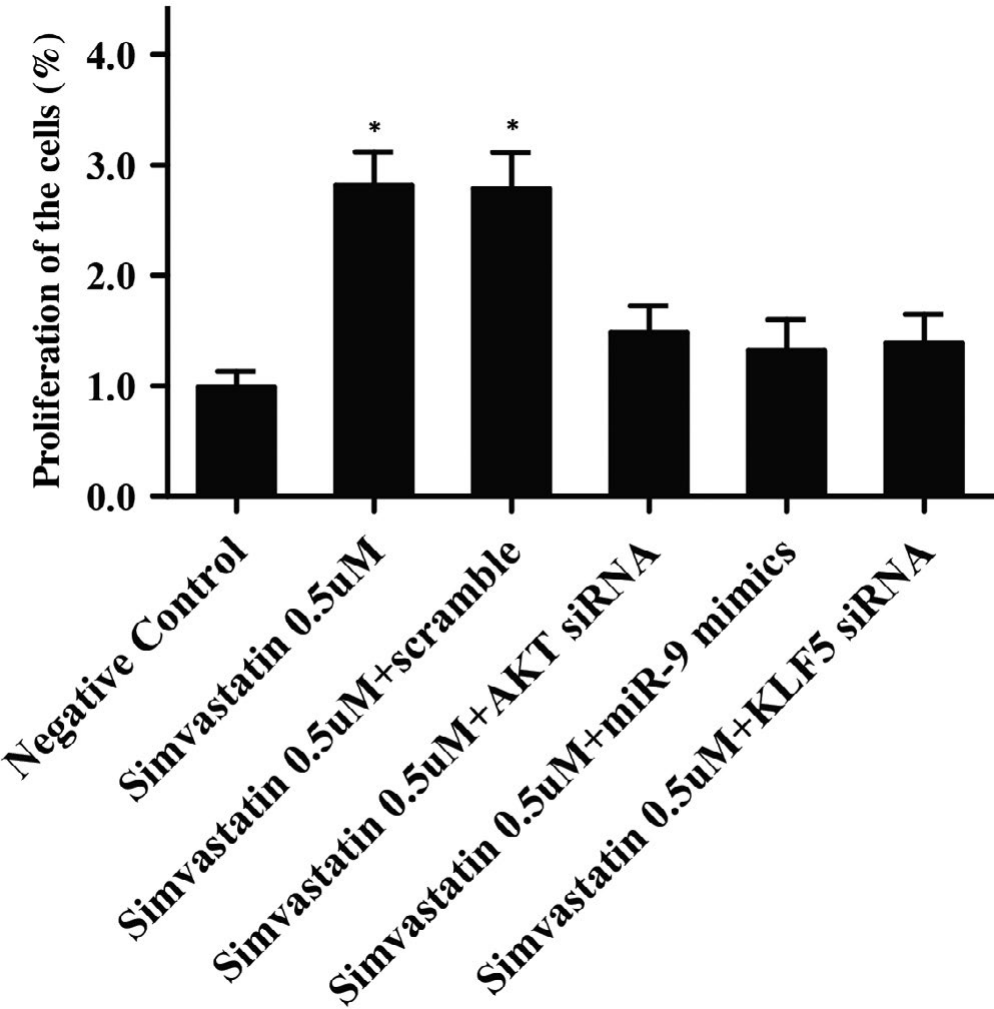
The effect of simvastatin, KLF5, AKT and miR‐9 on the proliferation of pulp stem cells (Asterisk *p* value < 0.05 vs. negative control group, untreated cells were referred as the control group)

In order to examine the effect of simvastatin, miR‐9, KLF5 and AKT on the proliferation of pulp stem cells, a CCK‐8 assay was performed, as shown in Figure 7, the cells treated with 0.5 μM simvastatin dramatically enhanced proliferation than control. On the contrary, cells transfected with 0.5 μM simvastatin plus miR‐9 mimic, KLF5 siRNA and AKT siRNA displayed a similar proliferation rate compared with negative control, suggested that simvastatin, KLF5 and AKT induced proliferation of pulp stem cells, whilst miR‐9 inhibited pulp stem cells proliferation.

## 4 DISCUSSION

In this study, we found that simvastatin significantly enhanced the proliferation of pulp stem cells, but this effect was completely abolished by LY294002 (an inhibitor of PIK3). Then, we performed western‐blot analysis and RT‐qPCR to measure PI3K, AKT and p‐AKT levels of cells following treatment with different concentrations of simvastatin and revealed that simvastatin upregulated the expression of PI3K dose‐dependently, and simvastatin upregulated p‐AKT expression level in a concentration‐dependent manner, whilst had no effect on the level of AKT. Furthermore, we found that simvastatin dose‐dependently enhanced the expression of PI3K and p‐AKT, whilst LY294002 completely abrogated the activation of p‐AKT induced by simvastatin, by contrast, simvastatin dose‐dependently reduced the expression of miR‐9.

A lot of studies have investigated abnormal miR‐9 expression in a range of cancers, indicating that miR‐9 could function as a tumour suppressor or as an oncogene. Increased expression of miR‐9 was found in endometrial cancer, primary brain tumours and Hodgkin's lymphoma, whereas decreased expression of miR‐9 was reported in gastric carcinoma, hepatocellular, ovarian, non‐small‐cell lung, colorectal and cervical cancer.^20^, ^21^, ^22^, ^23^, ^24^, ^25^, ^26^, ^27^ Reduced expression of miR‐9 was regarded as a marker for poor survival in acute lymphoblastic leukaemia, lung squamous cell carcinoma and cervical cancer, and it was also related to the onset of metastatic cancer.^25^, ^27^, ^28^, ^29^ It has been identified that simvastatin reduced the expression of miR‐9, miR‐21, miR‐132 and miR‐369‐3p in rat BMSCs.^18^ The expression of PI3K in BMSCs is modulated either by p‐AKT or miR‐9.^18^ These proteins may be associated; thereby, it has been measured that the expression of miR‐9 in BMSCs following treatment with different concentrations of simvastatin and LY294002.^18^ When compared to p‐AKT upon exposure to various concentrations of simvastatin, miR‐9 expression revealed an opposing trend, and miR‐9 expression was promoted by LY294002. These findings indicate that miR‐9 expression is regulated by p‐AKT.^18^ In this study, we search the online miRNA prediction tool miRanda, and found that KLF5 was the direct target of miR‐9 and miR‐9 mimic obviously inhibited the wild‐type KLF5 3’UTR luciferase activity, but not that of mutant KLF5 3’UTR.

The AKT/PI3K signalling pathway is a significant trigger of cell biology. Simvastatin promotes the phosphorylation of AKT in a number of cell types, including endothelial cells and EPCs.^30^ The culture of THP‐1 cells with simvastatin controls IL‐1b secretion and triggers the phosphorylation of AKT.^31^ In vascular smooth muscle cells, suppression of RhoA by simvastatin triggers elevated AKT phosphorylation.^32^ P‐AKT triggers the neuroprotective functions of simvastatin. Nevertheless, studies reveal that AKT phosphorylation can also be suppressed by simvastatin. In cultured aortic smooth muscle cells, pre‐treatment with simvastatin reduces AKT phosphorylation elicited by TNF‐a.^33^ In C2C12 myotubes, simvastatin results in compromised AKT phosphorylation and leads to decreased protein synthesis.^34^ Simvastatin has complicated roles in the AKT /PI3K signalling pathway in a variety of cell types. It has been identified that phosphorylation of AKT elevated with augmenting concentrations of simvastatin.^18^ In this study, we found that simvastatin dose‐dependently upregulated KLF5.

Meanwhile, we explored that the level of KLF5 in cells was much lower following the transfection with miR‐9 and KLF5 siRNA, and the level of AKT mRNA in cells was significantly inhibited following the treatment with AKT siRNA than control, whilst miR‐9 had no effect on the level of AKT mRNA. Finally, using the CCK‐8 assay, we proved that simvastatin, KLF5 and AKT promoted the proliferation of pulp stem cells, whilst miR‐9 attenuated pulp stem cells proliferation.

*Krüppel*‐*like factor 5* (*KLF5*, also identified as *BTEB2*, which is situated at the q21 band of human chromosome 13 (13q21) and encodes a basic transcription factor, comparative genomic hybridization studies revealed that it was centred in the second most commonly deleted chromosomal area in human prostate cancer.^35^ There were data revealing that *KLF5* commonly remains inactivated during carcinogenesis in humans and hence could be a tumour inhibitor gene, and several functional investigations actually back up a tumour inhibitory role of KLF5. For instance, KLF5 can inhibit the tumorigenesis of human prostate cancer cell lines in nude mice and suppress the proliferation of epithelial cells such as some cancer cell lines in culture.^35^, ^36^ Moreover, KLF5 enhances the self‐renewal of ESC and maintains ESC under undifferentiation.^37^ Recently, it has been shown that depletion of KLF5 substantially inhibits the tumour growth, proliferation and survival of basal TNBC cells in vivo.^38^ Bialkowska et al.^39^ found two small molecules that inhibited the expression of KLF5 expression and substantially suppressed the proliferation of colorectal cancer cells. Intriguingly, depletion of KLF5 triggers differentiation of mouse embryonic stem cells and inhibits proliferation and survival of intestine stem cells.^40^ Accordingly, the depletion of KLF5 substantially reduces CSC in HCC1937^41^

In the dental pulp, KLF5 is recognized to have a significant role in stemness, cell differentiation and proliferation. Ema (2010) indicated that KLF5 had pluripotent effects in embryonic stem cells and inhibited the expression of marker genes for differentiation.^42^ Recently, the reports have shown that KLF5 can function as a critical controller of cell differentiation including epithelial cells and adipocytes.^43^ It has been shown that KLF5 was abundantly present in the odontoblast‐like cells, and knock‐down of KLF5 substantially reduced odontoblastic differentiation of DPCs, reveal that KLF5 involves in the formation of reparative dentine and might also be obligatory for differentiation of odontoblast of DPCs.^44^ It has been confirmed in the expression of KLF5 in the differentiation of odontoblast of DPCs during the formation of reparative dentine.^44^

## 5 CONCLUSION

These findings suggested simvastatin could promote the proliferation of pulp stem cells, possibly by suppressing the expression of miR‐9 via activating the PI3K/AKT signalling pathway, and the downregulation of miR‐9 up‐regulated the expression of its target gene, KLF5, which is directly responsible for the enhanced the proliferation of pulp stem cells.

## CONFLICT OF INTEREST

The authors declare that they have no competing interests.

## AUTHOR CONTRIBUTION

**Jinghui Wang:** Conceptualization (equal); Data curation (equal); Formal analysis (equal); Validation (equal); Writing‐original draft (equal). **Dangen He:** Data curation (equal); Methodology (equal); Resources (equal); Supervision (equal); Writing‐original draft (equal).

## Data Availability

The datasets used and/or analysed during the current study are available from the corresponding author on reasonable request.
